# Predisposition to the Use/Non-Use of Mobility Aids in People with Neurological Impairment

**DOI:** 10.3390/healthcare14070825

**Published:** 2026-03-24

**Authors:** Estíbaliz Jiménez Arberas, Thais Pousada García, Feliciano Francisco Ordoñez Fernández

**Affiliations:** 1 Faculty Padre Ossó, University of Oviedo, 33008 Oviedo, Spain; feliciano@facultadpadreosso.es; 2 In-DIV Research Group, Faculty of Education and Humanities, UNIR (University International of La Rioja), 26006 Logroño, Spain; thais.pousada@unir.net; 3 Psychology Department, Faculty of Health Sciences, UNIR (University International of La Rioja), 26006 Logroño, Spain

**Keywords:** assistive technology, assistive product, patient reported outcome measures, mobility limitation, non-use, interruption

## Abstract

**Background/Objectives**: Assistive technologies are commonly used as a compensatory strategy for individuals with neurological conditions. However, several negative factors have been associated with their use, leading to their non-use or interruption. Therefore, the aim of the present study was to examine the potential of the Assistive Technology Device Predisposition Assessment (ATD-PA) as an outcome measure to identify psychosocial and user-perceived factors associated with the non-use or interruption of assistive technology, particularly mobility devices. **Methods**: A descriptive, cross-sectional and non-experimental design was employed, as no variables were manipulated. The sample was selected using non-probability convenience sampling and consisted of 80 participants, of which 14 participants discontinued or interrupted the use of assistive technology. An ad hoc sociodemographic questionnaire was administered, along with the Assistive Technology Device Predisposition Assessment, based on the Matching Person and Technology (MPT) model. **Results**: Factors related to non-use or interruption appeared to be associated with higher perceived levels of global health, self-care, and physical well-being. Findings from the ATD-PA, used as an indicator of subjective satisfaction, showed strong associations between the perceived level of loss and the need for assistive technologies in domains such as comfort, self-care, and general health (r = 0.72–0.90). The perceived benefit of the device was closely linked to knowledge of its use, safety, fit with personal habits, and perceived capability and stamina (r = 0.69–0.94). Comfort using the device was mainly reported in familiar environments such as with family and friends. In contrast, comfort in broader community contexts did not demonstrate meaningful associations. **Conclusions**: Findings are consistent with Lauer’s model of non-use and highlight the importance of psychosocial determinants such as perceived health, safety, support, and contextual comfort in understanding the interruption or non-use of assistive technology, in line with the International Classification of Functioning, Disability and Health framework. The ATD-PA shows potential as an outcome-oriented tool to support follow-up and the early identification of risk factors for non-use. Longitudinal studies are needed to better understand usage patterns over time. In Spain, the lack of standardized outcome evaluation protocols and systematic follow-up processes underscore the need for structured monitoring strategies in assistive technology provision.

## 1. Introduction

According to the Survey on Disability, Personal Autonomy and Situations of Dependency 2020, carried out by the National Institute of Statistics of Spain, there are 4.38 million people in the country declared to have a disability, of whom 58.6% (2.57 million) are women. In this survey, a person with a disability is considered as having a recognized degree of disability equal to or greater than 33% [1]. This degree is determined through a standardized technical assessment conducted by multidisciplinary teams. The evaluation integrates impairments in body functions and structures, activity limitations, participation restrictions, and contextual factors, in line with the biopsychosocial model of functioning. The final percentage reflects the interaction between the individual’s health condition and environmental barriers or facilitators [2]. In both sexes, these people with disabilities show more limitations in mobility [3]. According to the World Health Organization (WHO), neurological diseases are the leading cause of disability and the second leading cause of death in the world, with one out of every three people affected by this type of disease [4]. Their incidence is progressively increasing as a direct consequence of increased population life expectancy, constituting a major public health concern. Accordingly, the objectives extend beyond the survival of affected individuals to include the reduction in health loss associated with disability, with an emphasis on promoting function and independence [5,6].

One of the most commonly used strategies to mitigate, neutralize or compensate for limitations in functional mobility occupation are Assistive Technologies (AT). These, according to ISO standard Assistive Products Classification and terminology (ISO 9999:2022) [7], are “any product (including devices, equipment, instruments, and software) specially manufactured or commercially available, that optimizes a person’s functioning and reduces their disability”; this definition was made in line with the International Classification of Functioning, Disability and Health (ICF) [8]. Therefore, AT are included within the ICF environmental factors, and can be considered a barrier or a facilitator according to this classification. In Spain, the healthcare system is characterized as a public, tax-funded, universal, decentralized model that provides services free of charge at the point of delivery. Within this framework, the National Health System includes an orthoprosthetic benefits catalog, which is accessible to all individuals with a confirmed medical diagnosis requiring such products. Some of the AT commonly prescribed to people with neurological conditions are covered under this orthoprosthetic provision, including surgical implants (e.g., cardiac and vascular implants or implantable Holter devices) and external orthoprostheses (such as prostheses and both manual and powered wheelchairs). Although funding does not necessarily cover 100% of the cost in all cases, a substantial percentage of the total expense is generally financed by the public health system. If the AT they need is not included in this catalog, people usually acquire it in orthopedics or loan banks, although these are the least prevalent [9,10,11]. There are a variety of AT for activities and participation relating to personal mobility and transportation: electric wheelchairs, manual wheelchairs, canes and crutches, walkers, mass transit vehicles, mopeds and motorcycles. Although acquiring AT seems the best strategy, it has limitations such as the non-use of AT, non-adherence or abandonment of the AT. Although there are specific evaluation models and tools for AT, there is no specific model for non-use [12]. However, there are several specific assessment tools for outcomes in AT that have been used to evaluate AT non-use/interruption, such as the Psychosocial Impact of Assistive Devices [13,14].

In addition, other tools that come from specific models such as the Matching Person and Technology (MPT) tool, which explains concepts such as non-use, abandonment, and discontinuance, are also applied [15]. This model includes non-use being the result of a mismatch between the user and the technology, which may be due to insufficient training, inadequate device selection or a lack of customization [16]. This model includes several proprietary assessment tools, including the Assistive Technology Device Predisposition Assessment (ATD-PA), which was designed to support decision-making processes regarding AT by systematically considering key factors associated with their use and non-use during the selection phase. Its purpose is to facilitate a comprehensive and user-centered evaluation, incorporating aspects that may influence long-term adoption, satisfaction, and functional outcomes, thereby promoting more appropriate and sustainable prescription practices. Several previous studies have employed the ATD-PA to explore psychosocial factors influencing AT outcomes across diverse disability populations. Early validation work conducted within the MPT framework demonstrated its utility in adolescents and adults with various physical and cognitive disabilities, supporting its role as a structured tool to examine person–technology fit [15]. In these contexts, the ATD-PA was primarily used to identify factors associated with successful technology adoption and continued use. Similarly, research involving adults with spinal cord injury has applied the ATD-PA to examine the relationship between psychosocial predispositions and long-term device utilization. Findings from these studies suggest that perceived competence, expectations, and emotional responses to AT are linked to adherence and satisfaction outcomes, reinforcing the instrument’s relevance in rehabilitation settings [17,18].

This model and its tools have been little used in Spain, especially in terms of measuring the results of non-use/interruption in AT. The aim of this study was to examine the potential of the ATD-PA as an outcome-oriented tool for evaluating non-use and interruption patterns in AT utilization. Specifically, the study sought to analyze whether psychosocial, functional satisfaction, and user-perceived factors measured by the ATD-PA could help identify determinants associated with the continued use, discontinuation, or interruption of assistive technology, particularly in the context of mobility devices.

## 2. Materials and Methods

### 2.1. Design

This is a descriptive cross-sectional and non-experimental design since no variables were manipulated, with non-probabilistic convenience sampling.

### 2.2. Participants

Participants were recruited through convenience non-probability sampling from a network of NGOs and 13 rehabilitation centers specializing in neurological conditions. Participants were recruited using a snowball sampling strategy across multiple non-governmental organizations (NGOs) and rehabilitation clinics located in rural areas rather than urban centers. These settings were selected because they represent common service delivery contexts for AT provision in Spain. Given the absence of a comprehensive registry and the difficulty in accessing this population, the study was designed as an exploratory cross-sectional study with a convenience sample. The participating centers were identified through direct professional contact with the principal investigator and primarily involved occupational therapists, as this professional profile typically holds responsibilities related to the assessment, selection, and training of AT.

Eligibility criteria included: (1) a confirmed diagnosis of a neurological condition; (2) cognitive performance within normal limits (MMSE > 27); (3) age 18 years or older; and (4) current ownership or use of a mobility assistive device for personal mobility. Based on these criteria, a final sample of 80 participants was obtained. All individuals provided written informed consent, and data handling complied with current data protection and confidentiality regulations. Although a total pool larger than the final sample was initially approached, approximately 25% declined participation, mainly due to concurrent involvement in other research projects.

This study was approved by the relevant Research Ethics Committee (reference code 2021.010). All participants provided written informed consent prior to inclusion. The study was conducted in accordance with the ethical principles outlined in the Declaration of Helsinki and its subsequent amendments, ensuring confidentiality and voluntary participation throughout the research process.

The overall sample consisted of 44 men (55%) and 36 women (45%). At the time of assessment, 66 participants (82.5%) reported continued use of their assistive technology, whereas 14 participants (17.5%) indicated non-use or discontinuation. Within the non-use group, 57.1% were male and 42.9% female, while among users, 54.5% were male and 45.5% female, indicating a comparable sex distribution between groups. The mean age of the total sample was 59.44 years (SD = 16.6).

In the subgroup reporting non-use or discontinuation, most participants had a level of higher education (64.3%), followed by secondary education (28.6%) and primary education (7.1%). All individuals in this subgroup lived in their own homes. Regarding clinical diagnosis, the non-use group was predominantly composed of participants with stroke (50%), followed by neurodegenerative conditions (21.4%) and neuromuscular disorders (21.4%). A similar diagnostic distribution was observed among participants who continued to use their assistive technology, with stroke accounting for 50%, neurodegenerative conditions for 27.3%, and neuromuscular disorders for 10.6%.

For the interpretation of the results, the descriptive data of the variables are provided: time elapsed since diagnosis (the data are in days): min. = 3; max. = 720; mean = 113; SD = 142; and time of use of the AT: min. = 1; max. = 300; mean = 58.67; SD = 74.

### 2.3. Instruments

#### Ad Hoc Sociodemographic Questionnaire

The research group developed a questionnaire to collect data on personal factors, social factors, environmental factors and features of participants’ mobility assistive devices.

Assistive Technology Device Predisposition Assessment (ATD-PA) [17,18]: Helps guide AT decisions by considering important use and non-use factors during selection. The questionnaire is used as a measure of subjective satisfaction with the person’s current achievements in a wide variety of functional areas (ATD-PA_A; section A—9 items), asks users to prioritize those aspects of their lives where improvement is most desired (ATD PA_B; section B—12 items), provides a profile of users’ psychosocial characteristics (ATD PA_C; section C—33 items), and asks users about their perspective on 12 aspects related to the use of a given AT. Each item was rated on a five-point Likert scale (1–5), with higher scores reflecting higher perceived satisfaction. The Spanish version of the ATD-PA, included within the MPT assessment framework, was used in this study [18]. The instrument is publicly available and fully described in the MPT manual. Previous research on the ATD-PA has examined its psychometric properties, including inter-rater reliability, internal consistency, and criterion validity. In addition, multiple studies have applied the MPT framework as an outcome measure to evaluate assistive technology use and person–technology fit in diverse populations. The ATD-PA was administered by a researcher who participated in the Spanish adaptation of the instrument and has extensive experience with the MPT assessment framework, particularly with the ATD-PA. The administration time varied according to participant characteristics and the heterogeneity of functional conditions, ranging approximately between 25 and 60 min.

### 2.4. Procedure

Data were collected at a single baseline time point; therefore, the study follows a cross-sectional design. Six months later, participants were contacted only to determine their AT use status (continued use, interruption, or non-use), without any additional reassessment. Follow-up information could not be obtained in six cases for reasons unrelated to the study, and one participant had died.

### 2.5. Data Analysis

The SPSS program was used, along with Minitab 21, 2024 [19,20,21,22].

The data analysis was carried out in two distinct phases. In the first phase, a Spearman correlation analysis (rho) was performed to detect possible relationships with the non-use variable and was completed with a linear regression analysis.

With the results obtained, a comparative analysis was performed to eliminate possible external variables that could interfere with the results obtained. Since these were ordinal variables, the nonparametric Mann–Whitney U statistic was used for the comparative analysis.

In the second phase of the study, correlograms were made with each block of variables, segmenting the sample between users who drop out and those who do not. This analysis allows us to visualize and compare the relationships of strength and linear correlations between pairs of variables, which makes it possible to determine which variables act in the non-use or interruption of AT and the existing relationships.

Minitab 21 (2024) [22] has been used for these analyses; these analyses allow us to see the correlation statistics identifying the strong relationships between variables.

Significance values *p* less than or equal to 0.05, and correlation values greater than rxy of >0.65, were used for the interpretation of the correlograms. The average value of the strength range of the relationship in a correlation is considered to be between 0.50 and 0.75; we have used this average value as the limit for the robustness of the data results.

The study concludes with a correlational analysis between the ATD-PA variables and the total scores of the PIADS subscales. Only these subscales were included, as our previous study identified them as having a statistically significant relationship with the interruption or non-use of AT [13].

Given the normality characteristics of the ATD-PA scale variables in Parts A, B, and D, Pearson’s correlation coefficient was used to examine their relationships with the PIADS subscale scores. In contrast, Spearman’s rank correlation was applied for the analysis of the items in Part C.

## 3. Results

The first phase starts with a multiple correlational analysis between the dropout variable and the rest of the study variables. Spearman’s analysis shows that only three variables are related to the dropout variable, *p* = 0.035 “I aspire to go to school or work”; *p* = 0.024 “I am often frustrated or overwhelmed”; *p* = 0.034 “I am often angry”. The rest of the variables do not obtain significant values.

This first analysis has detected the variables that have a relationship, but it is not possible to determine which values are those that maintain this relationship, so a comparative analysis was carried out to determine whether there are significant differences between users who drop out and those who do not, and what these differences are due to.

For the analysis of all the ATDP-A, ATDP-B and ATDP-C variables, since they are Likert-type variables, the nonparametric Mann–Whitney U comparative analysis was performed, and no significant differences were obtained in any of the variables analyzed. It can be concluded that both groups, dropouts and non-dropouts, belong to the same population, so there are no significant differences in the variables analyzed.

The analysis was further complemented by the results of correlogram analyses. First, the variables corresponding to the ATD-PA instrument were examined in the subsample of users who discontinued or interrupted the use of assistive technology.

**Figure 1:**
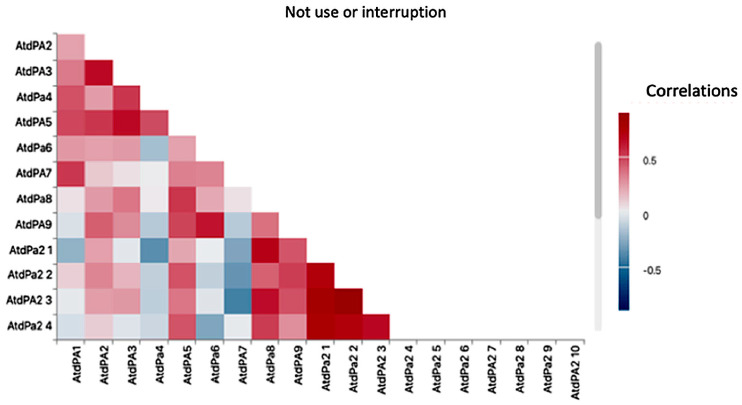
Correlogram of the study population based on ATD-PA_A and ATD-PA_B. Note: the correlation statistics that are established between the corresponding variables ATD-PA_A and ATD-PA_B, which range from variables 1 to 9 for ATD-PA_A and 1 to 11 for ATD-PA_B. On the *y*-axis, the ATD-PA_A and ATD-PA_B items are displayed, while the *x*-axis presents the ATD-PA_B items. Areas of higher correlation concentration are highlighted in red in the upper left and lower right quadrants, corresponding to the values emphasized inSection 3. ATD-PA2—hearing; ATD-PA3—speech; ATD-PA4—comprehension, recall; ATD-PA5—strength, physical endurance; ATD-PA6—use of lower body (hips, legs, feet); ATD-PA7—grasp and use of fingers; ATD-PA8—use of upper body (arms, shoulders, torso); ATD-PA9—mobility. ATD-PA 2.1—self-care; ATD-PA 2.2—comfort and physical well-being; ATD-PA 2.3—overall health; ATD-PA 2.4—freedom to go where you want.

In Figure 1, the existing relationships with non-use/interruption can be observed, with significant relationships of high concentration between ATD-PA_C and ATD-PA_B, 0.65 *p* ≤ 0.05, which would be those items that analyze the abilities in the areas of vision and hearing. More than 50% responded with values of 4 and 5 (Me = 4) in both cases. The result shows that the population of people with an excellent perception of their hearing and vision react towards not using/disrupting their use of AT by having a very positive feeling of their general health, self-care, comfort and physical well-being and having freedom to go where they want. This positive perception of the senses of sight and hearing is related to the responses of non-use of the AT.

Regarding the satisfaction variables in relation to different areas of the ATD-PA_B, other results can be observed in the previous figure. To give some examples, ATD-PA_B_2.2 “comfort and physical well-being” obtains a correlation value of 0.72 with ATD-PA_B_2.1 “personal care” data corresponding to high response values 4 and 5 (Me = 4). Also, the variable ATD-PA_B_2.3 “global health” obtains a correlation of 0.80 with ATD-PA_B_2.1 “personal care”, with ATD-PA_B_2.2 “comfort and physical well-being” showing a result of 0.90 and Me = 3.

Finally given its importance in relation to the mobility AT, the variable ATD-PA_B_2.4 “freedom to go wherever I want” has strong relationships with ATD-PA_B_2.1 (comfort and physical well-being), ATD-PA_B_2.2 (comfort and physical well-being) and ATD-PA_B _2.3 (global health); the rest of the variables do not reach strong correlation values, with a significance level > 0.05.

**Figure 2:**
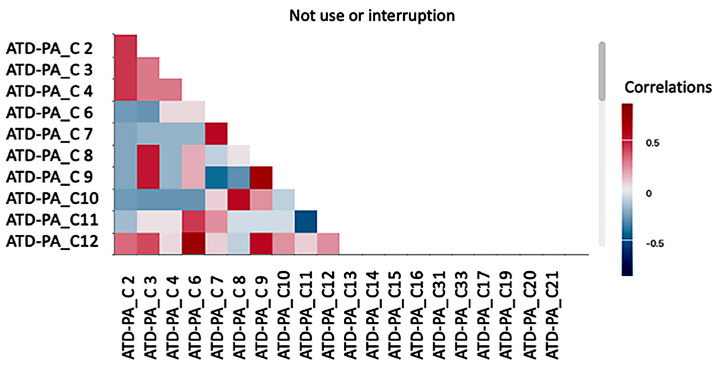
Correlogram based on the non-use/interruption of ATs and section C of the ATD-PA. Note: on the *y*-axis, the ATD-PA_C items are displayed, while the *x*-axis also represents the ATD-PA_C items showing inter-item correlation concentrations. Areas of higher correlation concentration are highlighted in red in the upper left quadrant, corresponding to the values emphasized in Section 3. No additional noteworthy correlations were observed. ATD-PA_C2—I have the support I want from my friends; ATD-PA_C3—I have support from my therapists/doctors, caregivers; ATD-PA_C4—I feel that people in general accept me; ATD-PA_C6—I aspire to go to school or work; ATD-PA_C7—I have many things I want to do; ATD-PA_C8—I do what my therapist/doctor asks without question; ATD-PA_C9—I see my therapist/doctor as a friend too; ATD-PA_C10—I am often frustrated or very overwhelmed; ATD-PA_C12—I am prepared to achieve my goals.

Figure 2 shows that it there are few correlations in relation to the non-use/interruption of AT; “I do what my therapist/doctor asks me to do without complaining” correlates with “I see my therapist as a friend”, with rxy = 0.68 and 72% of responses affirming this behavior; and “I am prepared to achieve my goals” with “I feel that people in general accept me”, with a percentage above 70% of the responses affirming these feelings.

**Figure 3:**
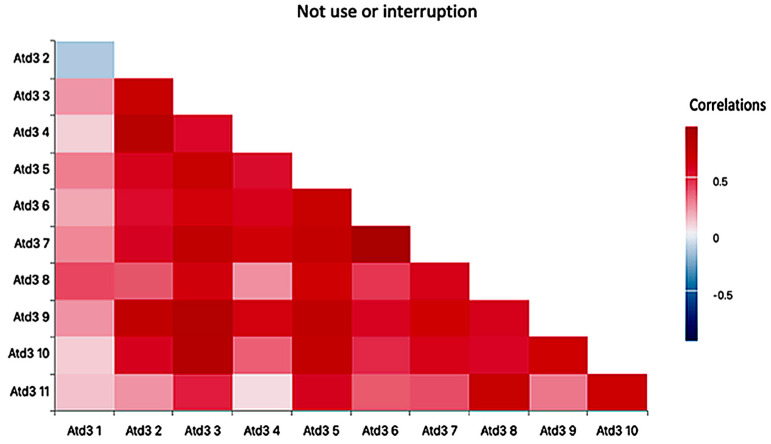
Correlogram based on willingness to not use/disruption of As with the evaluation of devices used from the ATD-PA_D. Note: ATD-PA D1—The AT helps me achieve my goals; ATD-PA D2—The AT benefits me and improves my quality of life; ATD-PA D3—I am confident in how to use the AT and its features; ATD-PA D4—I feel safe using the AT; ATD-PA D5—The AT fits well with my abilities and habits; ATD-PA D6—I have the necessary capabilities and stamina to use the AT without discomfort, stress, or fatigue; ATD-PA D7—I have the support, assistance, and adjustments to successfully use the AT; ATD-PA D8—The AT physically fits in all environments (home, car); ATD-PA D9—I feel comfortable (and not self-conscious) using the wheelchair when I am with my family; ATD-PA D10—I feel comfortable (and not self-conscious) using the AT when I am with friends; ATD-PA D11—I feel comfortable (and not self-conscious) using the AT in the community.

In Figure 3, we observe the highest concentration of strong relationships with the non-use/interruption of the AT. On the *y*-axis, the ATD-PA_D items are displayed, while the *x*-axis also represents the ATD-PA_D items showing inter-item correlation concentrations. Higher correlation concentrations are highlighted in red, particularly along the diagonal, corresponding to the values emphasized in the Section 3. Additionally, a high concentration of correlations is observed in the upper left quadrant, as well as in grid cells (3,2), (3,3), and (2,3).

ATD-PA_C_3.3 “I am sure I know how to use the AT and its characteristics” correlates with the statement “The AT benefits me and improves my quality of life”, ATD-PA_C_3.2, rxy = 0.69. This manifests in a knowledge among respondents of the support that the product provides, and the conditions it allows it to develop.

ATD-PA_C_3.4 “I feel safe when using the AP” correlates with ATD-PA_C_3.2, rxy = 0.82. ATD-PA_C_3.5 “The AT fits well with my habits and customs” correlates with ATD-PA_C_3.3, rxy = 0.70.

ATD-PA_C_3.6 “I have the capabilities and stamina to use the PA without comfort, stress or fatigue” correlates with ATD-PA_C_3.5, rxy = 0.69.

ATD-PA_C_3.7 “I have the support, assistance, and adjustments to successfully use the PA” correlates with ATD-PA_C_3.3, rxy = 0.75; with ATD-PA_C_3.6, rxy = 0.73; and with ATD-PA_C_3.6, rxy = 0.94.

ATD-PA_C_3.9 “I feel comfortable (and not self-conscious) using theAT when I am with my family” correlates with ATD-PA_C_3.2, rxy = 0.76; with ATD-PA_C_3.3, rxy = 0.87; and with ATD-PA_C_3.5, rxy = 0.77.

ATD-PA_C_3.10 “I feel comfortable (and not self-conscious) using the AT when I am with friends” correlates with ATD-PA_C_3.3, rxy = 0.85; with ATD-PA_C_3.5, rxy = 0.73; and with ATD-PA_C_3.9, rxy = 0.65.

ATD-PA_C_3.11 “I feel comfortable (and not self-conscious) using the AT when I am at work or school” correlates with ATD-PA_C_3.8, rxy = 0.68; and with ATD-PA_C_3.10, rxy = 0.66.

All the participant variables scored positively, which is why in order to understand non-use/interruption it is important to highlight that item 12 “I feel comfortable (and not self-conscious) using the AT when I am in the community” does not appear, since it does not reach positive values or relationship values. In general, participants feel satisfied with the AT in environments that are perceived as close and familiar. We cannot affirm the same when the person is in social situations that are not considered close or familiar. The results of the final relationship analysis of the scales have established the following results (Figure 1): between the subscales competence, self-esteem and adaptability with the variables ATD-PA-A (1.1 to 1.9); B (2.1 to 2.11) and D (3.1 to 3.11) no significant relationships were found.

## 4. Discussion

Globally, stroke is a leading cause of death and the resulting disability generates a considerable social and economic burden in Europe. It represents the leading cause of disability in Spain, a situation that is also reflected in other high-income countries. The incidence of stroke is expected to increase by 41% by 2040, especially in people over 80 years of age [23,24,25]. Although access to AT is a human right according to the United Nations, the International Disability Alliance reports that, in the latest WHO report on access to AT globally, approximately 1 billion people in low- and middle-income countries who need AT do not have access to it [26]. In Spain, the National Health System (SNS) has several catalogs of orthoprosthetic products that are financed by the public health system.

It includes external prostheses, orthoses, wheelchairs, and other assistive devices. The prescriber is usually the rehabilitation physician, neurologist or traumatologist, but in the case of stroke, only a standard wheelchair is usually included; otherwise, the user themself usually acquires the AT that is usually recommended in both public and private services, although without evaluation. In the present study 17.5% (n = 14) discontinued use; previous studies found a similar percentage, with 17.9%, although we found other studies with a higher percentage [27,28,29,30,31].

The rate at which AT is not used can vary widely, from as low as 8% to as high as 75%, depending on the type of AT [32]. Phillips and Zhao [33] conducted a survey of adults across the U.S. with various disabilities and discovered that ambulatory aids had the highest abandonment rates, with an average of 66% for canes, braces, or walkers. Recent findings further reinforce this perspective. For example, research on mobility assistive devices (MADs) among older adults reports non-adherence rates of up to 75% and identifies self-esteem as the strongest predictor of non-adherence, explaining nearly half of the variance in use patterns. This evidence converges with our findings and strengthens the argument that psychological constructs, particularly those linked to self-perception and identity, play a decisive role in sustained use [34]. The present findings should be interpreted within established theoretical frameworks of AT outcomes, particularly the MPT model proposed by Scherer [17]. The MPT framework conceptualizes AT use as an interactional process involving personal characteristics, psychosocial dispositions, environmental conditions, and service delivery variables. The application of the ATD-PA is therefore theoretically congruent, as the instrument was specifically developed to operationalize psychosocial predispositions influencing technology use. These findings describe associations between the perceived degree of loss; the perceived need for AT in domains of comfort, physical well-being, self-care, and general health; and patterns of non-use or interruption [35], suggesting that the subjective appraisal of functional status plays a central role in mobility AT engagement. Notably, higher self-perceived health and independence were associated with interruption patterns in the observed sample. These findings describe an association that may reflect different user experiences with assistive technology rather than a direct explanation of discontinuation. One possible interpretation is that, for some users, the interruption of device use could be related to a recalibration of perceived needs as functional status changes. This perspective is consistent with dynamic models of disability and functioning. In addition, the correlation observed between interruption and perceived “freedom to go where he/she wants” may be interpreted within Lauer’s conceptual framework, particularly in relation to personal factors such as perceived functional loss and processes of disability acceptance [36]. The subjective perception of autonomy may mediate engagement with mobility devices, suggesting that perceived independence can achieve sustained use even in the presence of objective impairment.

Section C of the ATD-PA, addressing evolving needs and technology-related variables, underscores the complexity of psychosocial mediation in AT outcomes [37]. While several determinants identified herein are represented in Lauer’s model, including device complexity, usability, and mismatch with user needs [36], current evidence indicates that psychosocial dimensions warrant greater theoretical centrality. Variables such as mood, self-determination, motivation, and perceived social support have demonstrated predictive relevance across multiple AT domains [16]. These findings suggest that existing frameworks may benefit from theoretical refinement to more explicitly integrate psychosocial constructs alongside technological and environmental determinants. In addition, evidence from systematic reviews highlights that satisfaction with AT is not solely determined by user-related or device-related factors but is also strongly influenced by the quality of the service delivery process (SDP). Factors operating across nearly all stages of the SDP (including assessment, selection, training, and follow-up) have been shown to affect user satisfaction, usability, and long-term utilization, potentially contributing to underuse or non-use when inadequately addressed. Consequently, a client-centered approach throughout the service delivery process has been identified as a critical determinant of effective outcomes and user satisfaction, reinforcing the need to conceptualize assistive technology use within a multidimensional framework that integrates psychosocial, technological, and service-related variables [38].

The therapeutic relationship represents a particularly underexamined variable. Although therapeutic alliance is robustly associated with treatment adherence and clinical outcomes in health research [38], its relationship with AT interruption remains insufficiently theorized. The present findings show that strong therapeutic engagement did not uniformly correspond with sustained device use in the observed sample. This pattern suggests that some users who remain actively involved in rehabilitation services may still interrupt or discontinue device use. One possible interpretation is that more empowered users may exercise greater autonomy in decisions regarding AT use. However, this interpretation should be considered cautiously, and future longitudinal research would be needed to better understand how therapeutic engagement relates to assistive technology use trajectories over time.

It is also essential to situate psychosocial determinants within broader contextual and systemic structures. Environmental accessibility, social support, service delivery quality, training adequacy, follow-up mechanisms, and funding models have all been documented as influential determinants of AT retention [39]. Moreover, alternative explanatory pathways must be acknowledged. Functional recovery, evolving occupational roles, contextual transitions, device replacement, and practical constraints may independently account for non-use or interruption. Consequently, AT non-use or interruption should be conceptualized as a dynamic outcome arising from reciprocal interactions between individual appraisal, environmental affordances, systemic structures, and service processes. In this context, the terminology “non-use or interruption” is methodologically preferable to “abandonment,” as it avoids implying intentional rejection and better reflects the potentially transient and contextually mediated nature of AT engagement. This conceptual precision aligns with contemporary outcome research and supports a more nuanced theoretical interpretation.

On the other hand, the PIADS has already been used as an outcome measurement tool for dropout in hearing aids, electronic aids, and mobility AT but it was not developed with this objective in mind, unlike the ATD-PA. The self-esteem subscale seems to correlate with non-use with items related to being proactive or in terms of the therapeutic relationship. On the other hand, the adaptability subscale correlates inversely if the person has family support, challenges themself and has a good therapeutic relationship. The results of this study suggest that the Lauer and Smith model should be revised and expanded to focus on these psychosocial aspects, and not only those related to the person, context or the AT itself. This could be in line with the International Classification of Functioning, Disability and Health [8], as taken into consideration by the MPT model. Although the Lauer model encompasses most of the factors relating to the advance of technology and sociodemographic and cultural changes, an update of the state of the art could be carried out. Among the limitations of this study are the use of non-probability convenience sampling and the relatively small number of participants classified as non-users or discontinuers (n = 14), which may limit statistical power in subgroup analyses. This limitation partly reflects the practical challenges associated with recruiting individuals with neurological conditions; therefore, findings involving this subgroup should be interpreted with caution. Furthermore, participants were recruited from NGOs and rehabilitation centers, which may have introduced selection bias. Individuals still engaged with services are likely overrepresented, whereas those who have fully disengaged—potentially including individuals who have abandoned their AT—may be underrepresented. However, this recruitment approach is often unavoidable in studies involving people with disabilities, as third-sector organizations and rehabilitation services frequently constitute the primary and most feasible access points to this population. Another aspect that should be considered concerns the statistical comparison between completers and non-completers. The absence of statistically significant differences between groups should not be interpreted as evidence of equivalence, but only as a lack of statistically detectable differences. Another limitation relates to the use of the ATD-PA scale and to the cross-sectional design of the study. Because of this design, the temporal direction of the associations cannot be established. Consequently, psychosocial variables measured with the ATD-PA should not necessarily be interpreted as antecedents of assistive device discontinuation, as they may also reflect users’ experiences after a reduced use of the device.

As for future lines of research, rehabilitation services should consider developing systematic protocols to assess the reasons underlying the non-use of AT. In the Spanish context, the evaluation of AT outcomes is not routinely conducted using validated instruments, which limits the ability to identify factors associated with non-use. Future studies should also further explore the relationship between psychosocial factors and AT use trajectories. Since instruments such as the ATD-PA capture users’ perceptions and experiences with AT, it would be particularly relevant to examine how these perceptions evolve over time and how they interact with patterns of continued use or discontinuation. Longitudinal designs could help clarify whether psychosocial variables precede changes in device use or emerge as a result of users’ experiences after adoption. Understanding these dynamic relationships may contribute to identifying critical moments in the process of AT integration and to developing interventions aimed at promoting sustained and satisfactory use.

## 5. Conclusions

Mobility assistive technologies constitute effective interventions for enhancing functional performance and independence; however, their sustained use is strongly influenced by psychosocial factors. The findings of this study indicate that users’ self-perceptions of health, independence, and well-being are closely associated with decisions regarding the continued use or discontinuation of assistive technology. In this sense, non-use does not necessarily reflect device inadequacy, but rather a complex subjective appraisal of personal needs and autonomy.

Additionally, the results suggest that the therapeutic relationship plays a meaningful role in assistive technology decision-making. A positive therapeutic alliance may support users’ capacity to make autonomous and informed choices regarding the use or non-use of assistive devices, reinforcing the importance of relational and contextual factors in rehabilitation processes.

From a clinical perspective, the non-use or interruption of AT should not be interpreted solely as device failure, as some individuals reporting non-use also perceive high levels of global health, self-care, and physical well-being. These findings suggest that AT use is dynamic and closely linked to perceived needs rather than only functional limitations. Therefore, clinical practice should emphasize ongoing reassessment of users’ perceived health status, psychosocial factors, and evolving occupational needs. Incorporating routine follow-up and patient-centered evaluation may help ensure better alignment between AT and users’ real-life contexts.

Overall, these conclusions highlight the need to conceptualize assistive technology use as a dynamic, person-centered process, in which psychosocial dimensions are central. Integrating these factors into assistive technology assessment and follow-up may enhance alignment with users’ goals, improve long-term outcomes, and strengthen rehabilitation practice within contemporary models of functioning and disability.

## Data Availability

Data is unavailable due to privacy or ethical restrictions.

## Abbreviations

The following abbreviations are used in this manuscript:

AT	Assistive Technology
ATD-PA	Assistive Technology Device Predisposition Assessment
MPT	Matching Person and Technology
PIADS	Psychosocial Impact of Assistive Devices Scale
SDP	Service Delivery Process
